# Hsa_circ_0026628 promotes the development of colorectal cancer by targeting SP1 to activate the Wnt/β-catenin pathway

**DOI:** 10.1038/s41419-021-03794-6

**Published:** 2021-08-21

**Authors:** Xuexiu Zhang, Jianning Yao, Haoling Shi, Bing Gao, Haining Zhou, Yanzhen Zhang, Dongyao Zhao, Shilin Gao, Chunfeng Wang, Lianfeng Zhang

**Affiliations:** 1 Department of Gastroenterology, The First Affiliated Hospital of Zhengzhou University, No.1 Jianshe East Road of Erqi District, 450052 Zhengzhou, Henan China; 2 Department of General Surgery, The First People Hospital of Zhengzhou, 450004 Zhengzhou, Henan China

**Keywords:** Cancer, Cell biology

## Abstract

Circular RNAs (circRNAs) have been reported to play crucial roles in the progression of various cancers, including colorectal cancer (CRC). SP1 (Sp1 transcription factor) is a well-recognized oncogene in CRC and is deemed to trigger the Wnt/β-catenin pathway. The present study was designed to investigate the role of circRNAs which shared the same pre-mRNA with SP1 in CRC cells. We identified that hsa_circ_0026628 (circ_0026628), a circular RNA that originated from SP1 pre-mRNA, was upregulated in CRC cells. Sanger sequencing and agarose gel electrophoresis verified the circular characteristic of circ_0026628. Functional assays including CCK-8, colony formation, transwell, immunofluorescence staining, and sphere formation assay revealed the function of circ_0026628. RNA pull-down and mass spectrometry disclosed the proteins interacting with circ_0026628. Mechanistic assays including RIP, RNA pull-down, CoIP, ChIP, and luciferase reporter assays demonstrated the interplays between molecules. The results depicted that circ_0026628 functioned as a contributor to CRC cell proliferation, migration, EMT, and stemness. Mechanistically, circ_0026628 served as the endogenous sponge of miR-346 and FUS to elevate SP1 expression at the post-transcriptional level, thus strengthening the interaction between SP1 and β-catenin to activate the Wnt/β-catenin pathway. In turn, the downstream gene of Wnt/β-catenin signaling, SOX2 (SRY-box transcription factor 2), transcriptionally activated SP1 and therefore boosted circ_0026628 level. On the whole, SOX2-induced circ_0026628 sponged miR-346 and recruited FUS protein to augment SP1, triggering the downstream Wnt/β-catenin pathway to facilitate CRC progression.

## Introduction

Colorectal cancer (CRC) is the 2nd leading cause of cancer-related deaths (9.2% of the total cancer deaths), and the 3rd most commonly diagnosed cancer (10.2% of the total cases) in 2018^1^. Although great advancement has been achieved in CRC diagnosis and treatment, the mortality of CRC is still frustratingly high. Thus, it is in urgent need to explore the in-depth mechanisms underlying CRC occurrence.

Recently, circular RNAs (circRNAs) have been gradually uncovered to play crucial roles in cancer progression. It has been widely accepted that circRNAs are positive contributors in cell proliferation, migration, epithelial-mesenchymal transition (EMT), and stemness characteristic^2,3^. As a newly-identified noncoding RNAs (ncRNAs), circRNAs are widely expressed in diverse cell types, and more than 20,000 circRNAs have been found in eukaryotes^4^. CircRNAs are characterized by their covalently closed-loop structures without 5′ caps and 3′ poly (A) tails^5^. The closed-loop structure is generated from the joining of the upstream 3′ splice acceptor to the downstream 5′ splice donor^6^. CircRNAs are resistant to exonuclease owing to their special closed-loop structure, which makes circRNAs more stable than linear RNAs^7^. In general, circRNAs can be derived from exonic, intronic, and intergenic regions^8^. Currently, the most studied circRNAs are the most abundant exonic ones which mainly reside in the cytoplasm^9^.

Further, circRNAs have been widely reported to play their roles via the competitive endogenous RNA (ceRNA) pattern. As a typical post-transcriptional mechanism, ceRNA pattern refers to that circRNAs compete with messenger RNAs (mRNAs) for the binding of microRNAs (miRNAs), thus nullifying the suppression of miRNAs on mRNAs. For instance, circ_0005963 suppresses glycolysis and reverses oxaliplatin resistance via sponging miR-122 to elevate PKM2 in CRC cells^10^. Circ_0005100, derived from FMN2, promotes CRC tumor growth by its mediation on miR-1182/hTERT axis^11^. Circ_0009361 functions as the miR-582 sponge to alleviate CRC progression via upregulating APC2 and inhibiting Wnt/β-catenin signaling^12^. In addition, circRNAs have been identified to bind with RNA-binding proteins (RBPs) to affect gene expression at different levels including post-transcriptional level. Du et al.^13^ disclosed that circ-Foxo3 inhibits cell cycle progression through binding to CDK2 and p21. Circ-ZKSCAN1 binds with FMRP against CCAR1 complex to negatively regulate stemness in hepatocellular carcinoma^14^.

As a sequence-specific DNA-binding protein, Sp1 transcription factor (SP1) actively participates in intracellular gene transcription. Thus, SP1 is a crucial transcription factor for multiple genes. Aberrant expression of SP1 is deemed to promote the initiation and progression of human cancers including CRC^15^. Besides, SP1 is closely associated with the activation of Wnt/β-catenin signaling^16^. The Wnt/β-catenin pathway is a complex and fundamental pathway that covers several different transduction cascades. The canonical Wnt/β-catenin pathway transcriptionally activates target genes that are involved in cell proliferation and stem cell renewal^17^. Besides, the oncogenic role of the Wnt/β-catenin pathway in CRC has been commonly revealed. Jiang et al.^18^ pointed out that USP22 contributes to CRC cell stemness and decreases the sensitivity of CRC cells to chemoresistance via the Wnt/β-Catenin pathway. Gu et al.^19^ revealed that miR-532-3p restrains cell growth, migration, and EMT in CRC via disrupting ETS1/TGM2 axis-mediated Wnt/β-catenin signaling.

The present study sought to examine circRNAs, which shared the same pre-mRNA with SP1 (genomic position: chr12:53,773,979-53,810,226). We identified that circ_0026628 (genomic position: chr12:53776037-53803345) was most significantly upregulated in CRC cells. Also, we identified that circ_0026628 positively regulated SP1 to accelerate CRC progression. Furthermore, the present study illustrated how circ_0026628 regulated SP1 as well as the interplays between circ_0026628/SP1 and Wnt/β-catenin signaling in CRC, which might enrich the academic knowledge of CRC.

## Materials and methods

### Cell lines and reagents

Human normal colorectal mucosal cell line (FHC) and 4 human CRC cell lines (SW620, LOVO, SW480, SW116) were used for the study, and all of them were available from ATCC Company (Manassas, VA). DMEM (Invitrogen, Carlsbad, CA) supplemented with 10% FBS and 1% antibiotics were applied for cell culture under 5% CO_2_ at 37 °C. For treating SW480 and LOVO cells, 2 μg/ml of ActD and DMSO (Mock) were procured from Sigma-Aldrich (St. Louis, MO), while 3 U/μg of RNase R was bought from Epicentre Technologies (Madison, WI). LiCl (5 mg), an activator of the Wnt signaling pathway, was purchased from Med Chem Express (Monmouth Junction, NJ).

### Total RNA extraction and qRT-PCR

Total RNA extraction was completed using Trizol reagent (Invitrogen), and then PrimeScript™ RT reagent kit was utilized for cDNA synthesis as per the direction. Gene expression was detected by qRT-PCR with SYBR Premix Ex Taq II (Takara), and processed based on the 2^−^^ΔΔCt^ method after being standardized to GAPDH or U6. The primer sequences were shown in Supplementary Table 1.

### Plasmid transfection

The specifically designed shRNAs and NC-shRNAs were constructed by GenePharma (Shanghai, China) to silence circ_0026628, FUS, and SOX2. For overexpression, circ_0026628 sequence was inserted into pLCDH-ciR-vector (Invitrogen), and the full-length cDNA sequence of SP1 or SOX2 was cloned into pcDNA3.1 vectors (Invitrogen), with the corresponding empty vector as the negative control. In addition, miR-346-mimics/inhibitor and NC mimics/inhibitor were also acquired from GenePharma. All plasmids were subjected to transfection into LOVO and SW480 cells for 48 h by use of Lipofectamine 2000 (Invitrogen). The target sequence of sh-circ_0026628#1 was: 5′-CATTGGCACCCTGTGTGTGTA-3′, and that of sh-circ_0026628#2 was: 5′-CAGCCATTGGCACCCTGTGTG-3′.

### FISH

LOVO and SW480 cells were treated with 4% formaldehyde for fixing, rinsed in PBS, and dehydrated, followed by incubation with the circ_0026628-FISH probe (Ribobio, Guangzhou, China). After 3 h of hybridization, cells were counterstained with Hoechst dye and observed using a fluorescence microscope (Olympus Corp., Tokyo, Japan).

### Subcellular fraction

PARIS™ Kit (Invitrogen) was acquired for subcellular fraction assay in LOVO and SW480 cells. After centrifugation, cells were processed with cell fractionation buffer or cell disruption buffer to obtain indicated cytoplasmic or nuclear fraction, respectively. Circ_0026628 content in cell nuclei and cell cytoplasm was detected by qRT-PCR.

### Cell proliferation assay

Cell counting kit-8 (CCK-8, Dojindo, Rockville, USA) was employed for this assay as per the user guide. Briefly, cells (2 × 10^3^) were planted in each well of the 96-well plates. After overnight incubation, cells in the plates were further grown for 0, 24, 48, 72, and 96 h, followed by 1 h processing with CCK-8 solution. Then, the absorbance at 450 nm was tested and recorded for each well by a microplate reader Victor (Enspire 2300 Multilabel Reader, PerkinElmer, Singapore).

### Colony formation assay

Clonogenic cells were subjected to 14-day cell culture in 96-well plates, with 500 cells in each well. Colonies were then fixated in 4% formaldehyde and stained by 0.1% crystal violet for counting.

### Transwell migration assay

The processed CRC cells were re-suspended in a serum-free medium for seeding into the upper chamber of the transwell insert (Corning, Corning, NY). Then the lower chamber was supplemented with the complete culture medium. One day later, cells migrating to the bottom were fixed and then stained in 0.1% crystal violet. Finally, 5 fields were chosen randomly for the counting of migrated cells via a light microscope (Olympus Corp.).

### Wound-healing assay

The transfected CRC cells were seeded in 6-well plates adding with serum-free medium. Then, the wells were scratched using sterile pipette tips. Cell samples were washed in PBS for clearing the detached cells. The distance of wound healing was recorded at 0 and 24 h.

### Western blot

Cells were lysed in RIPA lysis buffer, and then total protein concentration was examined with a BCA kit. Samples were separated on 12% SDS-PAGE and shifted to PVDF membranes. Following, the membranes were probed with primary antibodies after culturing with 5% nonfat milk. The primary antibodies specific to GAPDH (loading control), E-cadherin, N-cadherin, Slug, Twist, OCT4, Nanog, SOX4, SP1, FUS, β-catenin, c-Myc, and SOX2, and the corresponding HRP-tagged secondary antibodies, were all obtained from Abcam (Cambridge, MA). Enhanced chemiluminescence (ECL) system was applied for the detection of band signals as instructed (Amersham Pharmacia, Piscataway, NJ).

### Immunofluorescence (IF) staining

CRC cells in culture slides were rinsed by PBS, fixed for 10 min, and sealed by 5% BSA. The primary antibodies specific to E-cadherin, N-cadherin, FUS, SP1 and β-catenin, as well as the matched secondary antibodies, were separately added for the IF assay. After staining by DAPI dye, cells were analyzed by the fluorescence microscope.

### Sphere formation assay

The processed CRC cells were planted into 96-well ultralow attachment plates (Corning) with the sphere medium at a density of 10 cells/well. After 3, 7, 10 days of growth, the cell clusters with a diameter of more than 50 mm were seen as spheres and were counted under the light microscope.

### RIP assay

Cell lysates were collected using RIP lysis buffer, and then mixed with anti-Ago2 antibody or anti-FUS antibody that was conjugated to magnetic beads. Groups with anti-IgG antibodies were seen as negative controls. After immunoprecipitation, RNA enrichment was quantified by qRT-PCR.

### RNA pull-down assay

Pierce Magnetic RNA-Protein Pull-Down Kit was acquired from ThermoFisher Scientific (Waltham, MA) for RNA pull-down assay. Cell extracts were mixed with the biotin-labeled RNA probes for circ_0026628 and magnetic beads. Samples were finally analyzed by western blot or qRT-PCR.

### Luciferase reporter assay

Circ_0026628 or SP1 fragments covering miR-346 binding sequences (wild-type or mutant) were prepared to acquire circ_0026628-WT/Mut and SP1-WT/Mut, which were then loaded into pmirGLO luciferase vectors to obtain indicated recombinant reporters. After that, SW480 and LOVO cells were co-transfected with the above recombinant reporters and miR-346 mimics (or NC mimics). In addition, cells were co-transfected with pcDNA3.1-SOX2 (or pcDNA3.1) and pGL3 luciferase vector containing SP1 promoter. After 48 h of transfection, the luciferase intensity was assessed by Luciferase Reporter Assay System (Promega, Madison, WI).

### Bioinformatics analysis

To predict the potential miRNAs interacting with circ_0026628 or SP1, starBase v2.0 (http://starbase.sysu.edu.cn/index.php) was applied as described^20^. Under the conditions of CLIP-Data ≥ 1 and Degradome-Data ≥ 0, 107 miRNA candidates were screened out to bind to circ_0026628. Meanwhile, according to the screening conditions of CLIP-Data ≥ 1, Degradome-Data ≥ 0, pan-Cancer ≥ 4, and program Num ≥ 1, a total of 229 miRNAs were suggested to target SP1. Then, after putting the above two subsets in Venn, 45 miRNAs shared between circ_0026628 and SP1 were identified.

### ChIP assay

The binding of SOX2 to the SP1 promoter was examined by ChIP assay in line with the protocol of EZ-ChIP™ Kit (Millipore, Billerica, MA). After fixing for 10 min, the cross-linking of DNA and protein was acquired, and then DNA was fragmented randomly by ultrasonic. Samples were precipitated with SOX2 or IgG antibody (negative control) overnight, and then the precipitated DNA fragments were collected for qRT-PCR analysis.

### Co-immunoprecipitation (CoIP)

Cell lysates collected by IP lysis buffer were subjected to all-night incubation with SP1 or β-catenin antibody overnight at 4 °C, and the mixture was vibrated at a constant speed. IgG antibody was used in control groups. The beads were added, and the precipitated proteins were collected for subsequent western blot analysis.

### Statistical analyses

The experiments in our study were bio-repeated three times. Results were all analyzed by PRISM 6 (GraphPad, San Diego, CA) and given as the means ± standard deviation (SD). Statistical analyses were processed by Student’s *t*-test or one‐way/two‐way analysis of variance (ANOVA), with *P* < 0.05 as the significant level.

## Results

### Circ_0026628 was upregulated in CRC cells

**Fig. 1:**
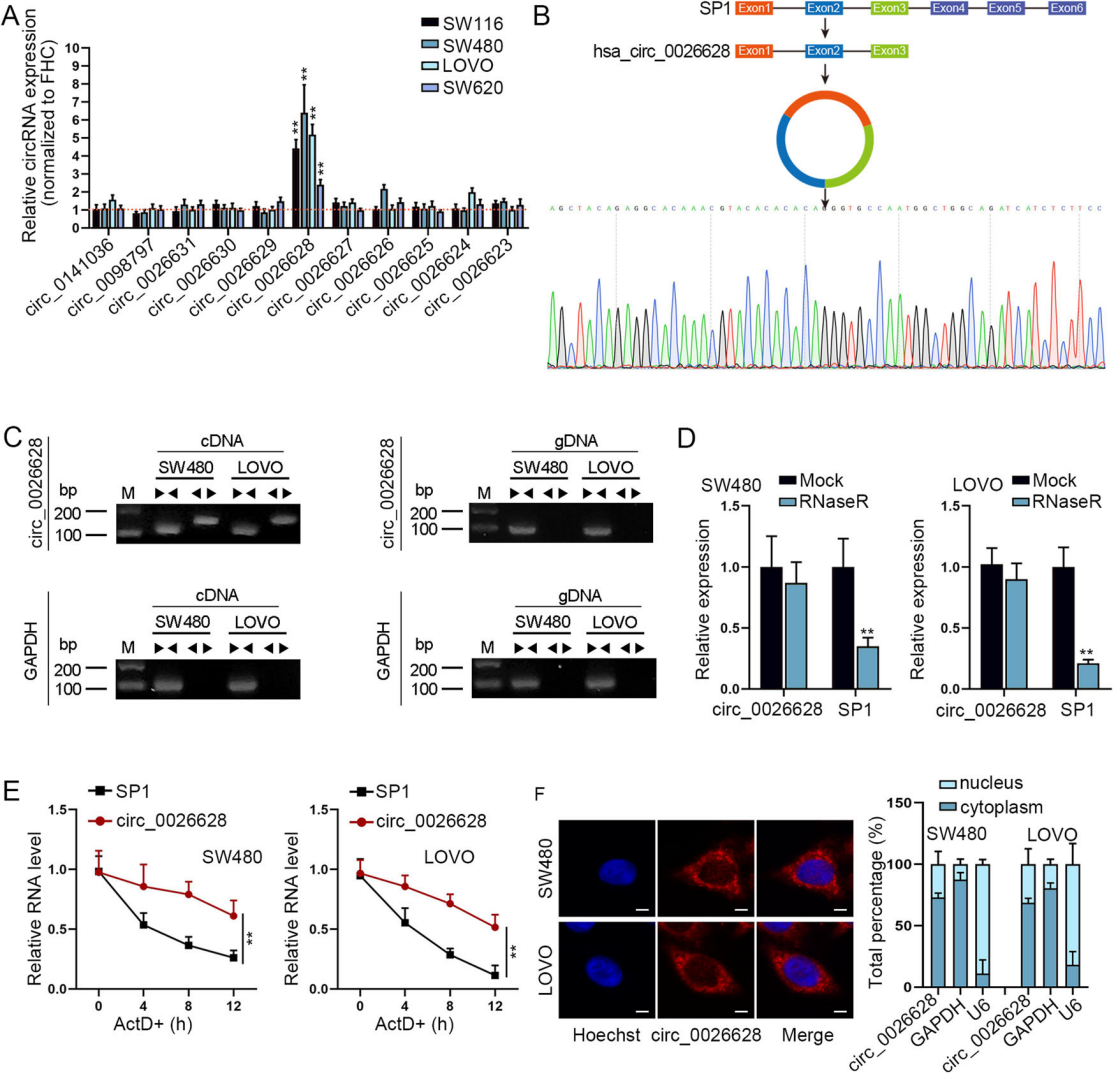
Circ_0026628 was upregulated in CRC cells. **A** qRT-PCR measured the expression profile of 11 SP1-derived circRNAs in CRC cells relative to the control FHC cells. One-way ANOVA. **B** The schematic diagram of the genomic position of circ_0026628 and the Sanger sequencing results for the splicing junction of circ_0026628. **C** Agarose gel electrophoresis validated the existence of circ_0026628 in CRC cells. **D**, **E** qRT-PCR measured the relative level of circ_0026628 and linear SP1 in CRC cells under the treatment of RNase R or ActD. Student’s *t*-test or two-way ANOVA. **F** FISH (scale bar: 20 μm) and subcellular fraction assays revealed the subcellular location of circ_0026628 in SW480 and LOVO cells. **P < 0.01.

Based on circBase^21^, 11 circRNAs which shared the same pre-mRNA with SP1 were identified. We then conducted a qRT-PCR analysis to detect the expression of these 11 circRNAs in 4 CRC cell lines and the normal control cell line (FHC). The results revealed that only circ_0026628 was significantly upregulated in 4 kinds of CRC cells than in the control cells (Fig. 1A). Since SW480 and LOVO cells exhibited the most significant upregulation of circ_0026628, they were used for the following assays. Next, we sought to verify the circular characteristic of circ_0026628. As demonstrated in Fig. 1B, circ_0026628 was spliced from exons 1 to 3 of SP1 and the Sanger sequencing results validated its splicing junction. Then, we designed convergent primers to amplify linear transcripts and divergent primers to amplify circular transcripts, with cDNA and gDNA as the templates. The results revealed that circ_0026628 was only amplified by divergent primers in cDNA while SP1 was amplified by convergent primers in both cDNA and gDNA (Fig. 1C), indicating that circ_0026628 possessed a circular structure. Following that, RNase R was applied to treat SW480 and LOVO cells, with the untreated cells (Mock) as controls. It was revealed that compared with linear SP1, circ_0026628 possessed with stronger resistance to RNase R treatment (Fig. 1D), which meant circ_0026628 was harder to be degraded than the linear SP1. Also, under the treatment of ActD, the level of linear SP1 was decreased faster than that of circ_0026628 as time went on (Fig. 1E), suggesting that circ_0026628 was more stable than the linear SP1. All the above results supported the circular characteristic of circ_0026628. Next, FISH and subcellular fraction assays were conducted to determine the subcellular location of circ_0026628. The results demonstrated that circ_0026628 mainly resided in the cytoplasm of CRC cells (Fig. 1F), implying the post-transcriptional role of circ_0026628 in CRC.

### Circ_0026628 promoted CRC cell proliferation, migration, EMT, and stemness

**Fig. 2:**
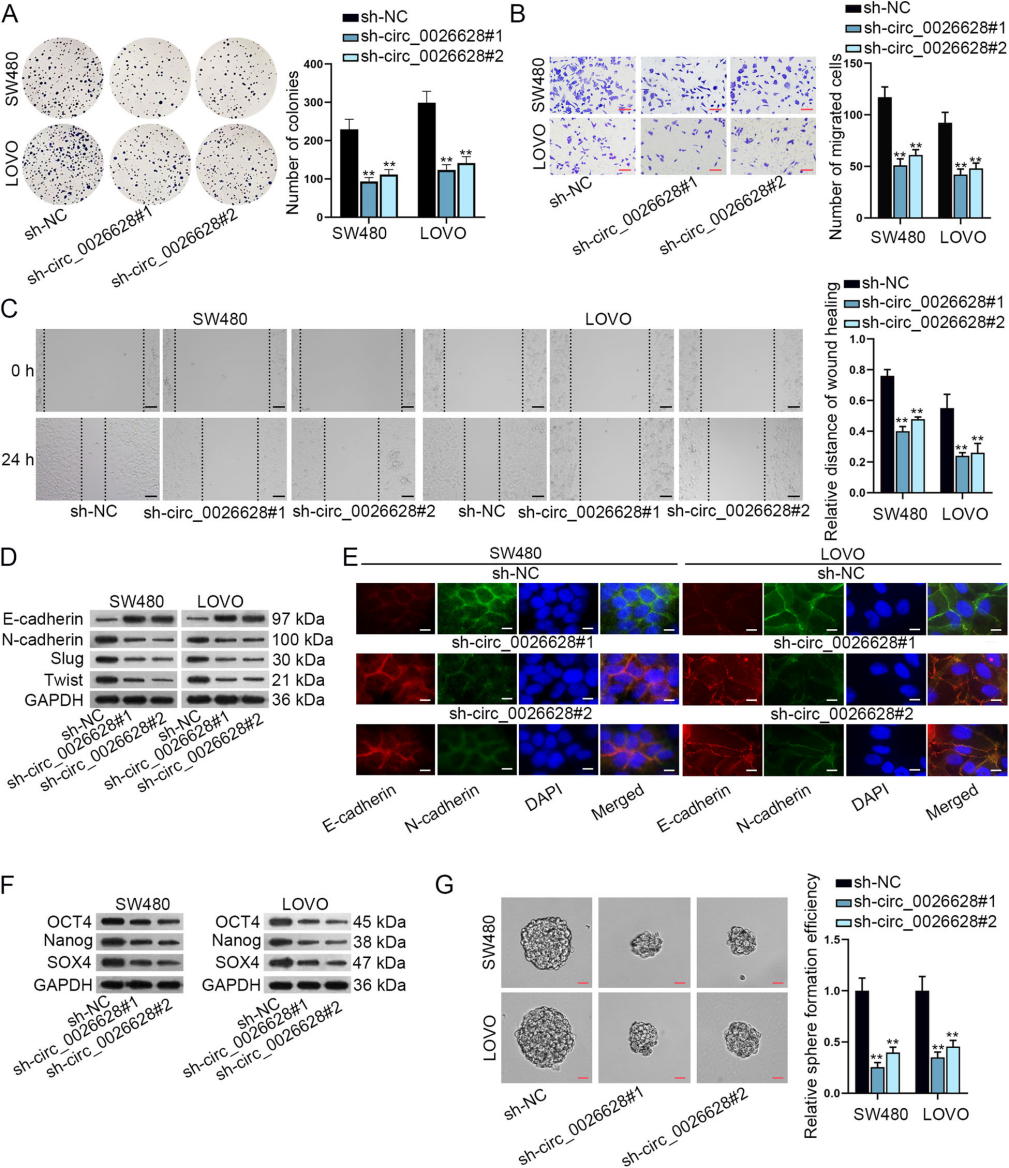
Circ_0026628 promoted CRC cell proliferation, migration, EMT, and stemness. **A** Colony formation assay examined the influence of silenced circ_0026628 on the proliferation ability of CRC cells. One-way ANOVA. **B**, **C** Transwell (scale bar: 180μm) and wound-healing assays (scale bar: 120 μm) revealed the migration ability of circ_0026628 silenced cells. One-way ANOVA. **D** Western blot determined the levels of EMT biomarkers including E-cadherin, N-cadherin, Slug, and Twist in circ_0026628 silenced cells. **E** IF staining assay (scale bar: 150 μm) analyzed the effects of silenced circ_0026628 on the positivity of E-cadherin and N-cadherin in CRC cells. **F** Western blot explored the expression of stemness biomarkers (OCT4, Nanog, and SOX4) in circ_0026628 silenced cells. **G** Sphere formation assay (scale bar: 150 μm) detected the sphere formation efficiency of circ_0026628 silenced cells. One-way ANOVA. **P < 0.01.

Subsequently, the function of circ_0026628 was examined in CRC cells. Before the loss-of-function assays, the depletion efficiency of circ_0026628 by sh-circ_0026628#1/2 was validated via qRT-PCR (Supplementary Fig. S1A). Importantly, it was proved that sh-circ_0026628#1/2 had no obvious impact on the expression of SP1 pre-mRNA (Supplementary Fig. S1B), indicating sh-circ_0026628#1/2 did not target SP1 but was special for circ_0026628. Then, we identified that silenced circ_0026628 reduced the proliferative ability of SW480 and LOVO cells (Fig. 2A and Supplementary Fig. S1C). Next, cell migration was assessed through transwell and wound-healing assays. The results revealed that loss of circ_0026628 impaired the migration ability of two CRC cells (Fig. 2B, C). Further, we analyzed the impact of circ_0026628 on EMT. It manifested that the absence of circ_0026628 significantly reduced the levels of N-cadherin, Slug, Twist and enhanced the expression of E-cadherin (Fig. 2D). Consistently, IF assay data also supported that circ_0026628 deficiency fortified the staining of E-cadherin and lessened the positivity of N-cadherin (Fig. 2E). Moreover, we also evaluated whether circ_0026628 affected the stemness of CRC cells. Notably, the levels of stemness biomarkers (OCT4, Nanog, and SOX4) were reduced in response to the knockdown of circ_0026628 (Fig. 2F). In addition, sphere formation assay results disclosed that the number and size of spheres were both decreased and the relative sphere formation efficiency was also remarkably reduced in CRC cells with circ_0026628 depletion (Fig. 2G and Supplementary Fig. S1D). In brief, circ_0026628 promoted cell proliferation, migration, EMT, and stemness in CRC.

### SP1 was required in circ_0026628-mediated biological functions of CRC cells

**Fig. 3:**
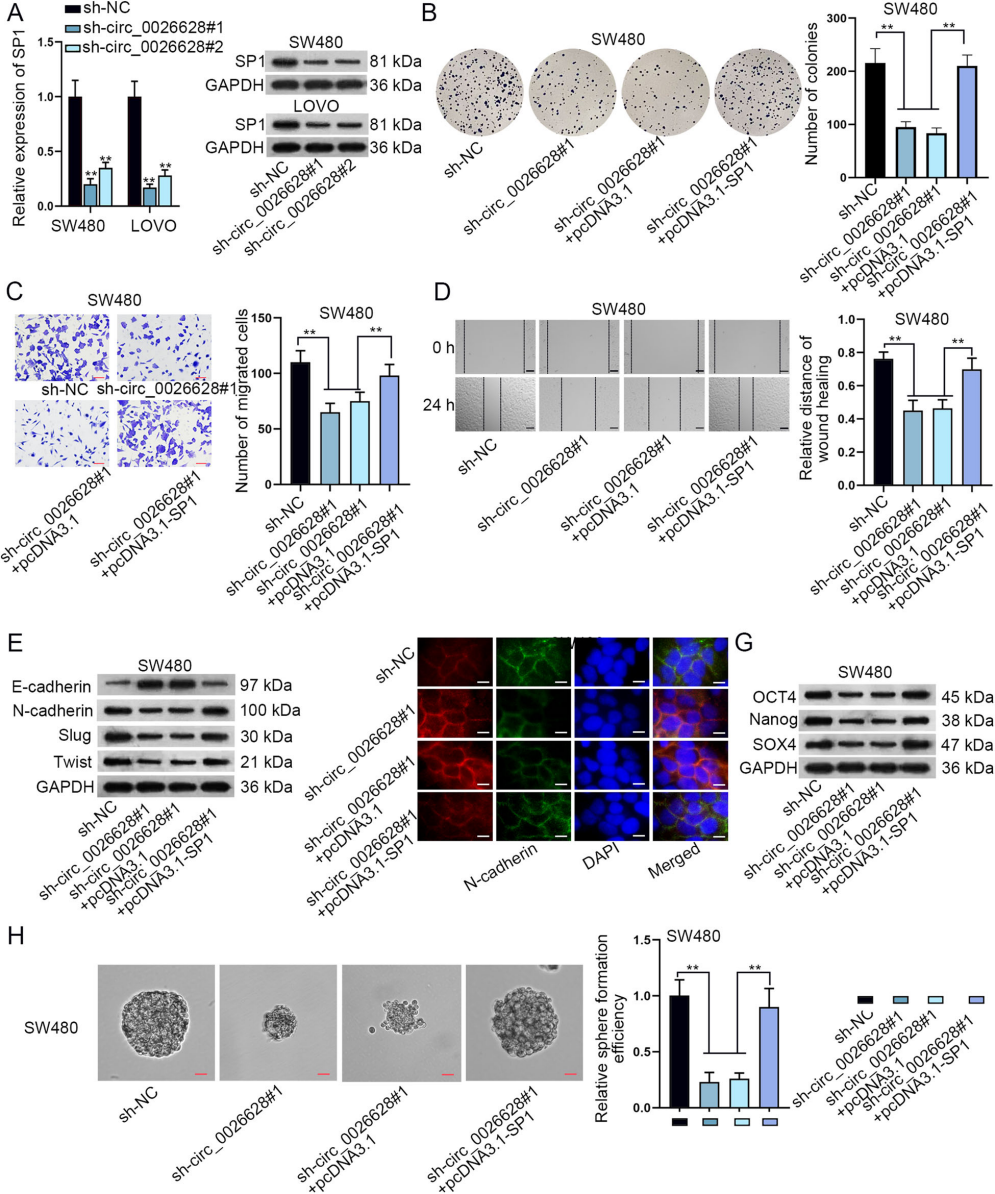
SP1 was required in circ_0026628-mediated biological functions of CRC cells. **A** qRT-PCR and western blot measured the influence of silenced circ_0026628 on SP1 expression in CRC cells. One-way ANOVA. **B**–**D**. Colony formation, transwell (scale bar: 180 μm) and wound-healing assays (scale bar: 120 μm) examined the rescue effects of SP1 overexpression on circ_0026628 deficiency hampered cell proliferation and migration. One-way ANOVA. **E**, **F** Western blotting and IF staining assay (scale bar: 150 μm) detected changes in EMT process in indicated CRC cells. **G**, **H** Western blot and sphere formation assay (scale bar: 150 μm) explored the stemness of CRC cells under different transfections. One-way ANOVA. **P < 0.01.

After we have verified the function of circ_0026628, we next sought to examine the underlying mechanism of circ_0026628 in CRC. We firstly detected the influence of circ_0026628 on SP1 expression. As was revealed in Fig. 3A, the mRNA and protein levels of SP1 were both decreased by silenced circ_0026628. Also, upregulated circ_0026628 caused an increase in SP1 expression (Supplementary Fig. S1E). Thus, rescue assays were conducted in SW480 cells to examine whether circ_0026628 affected CRC cell functions via targeting SP1. In advance of the rescue assays, the overexpression efficiency of SP1 was validated by qRT-PCR (Supplementary Fig. S1F). Then, the results of colony formation and CCK-8 assay proved that upregulating SP1 enhanced the hampered CRC cell proliferation due to circ_0026628 depletion (Fig. 3B and Supplementary Fig. S1G). Similarly, the outcomes of transwell and wound-healing assays revealed that SP1 upregulation completely rescued the suppressive effects of silenced circ_0026628 on cell proliferation and migration (Fig. 3C, D). Further, the inhibitory effects of downregulated circ_0026628 on EMT process were completely restored by upregulated SP1 (Fig. 3E, F). Besides, western blotting analysis of stemness markers and sphere formation assay data demonstrated that SP1 overexpression completely counteracted the effects of circ_0026628 deficiency on cell stemness (Fig. 3G, H). To sum up, circ_0026628 positively regulated SP1 expression to facilitate CRC cell proliferation, migration, EMT, and stemness.

### Circ_0026628 competed with SP1 for the binding of miR-346

**Fig. 4:**
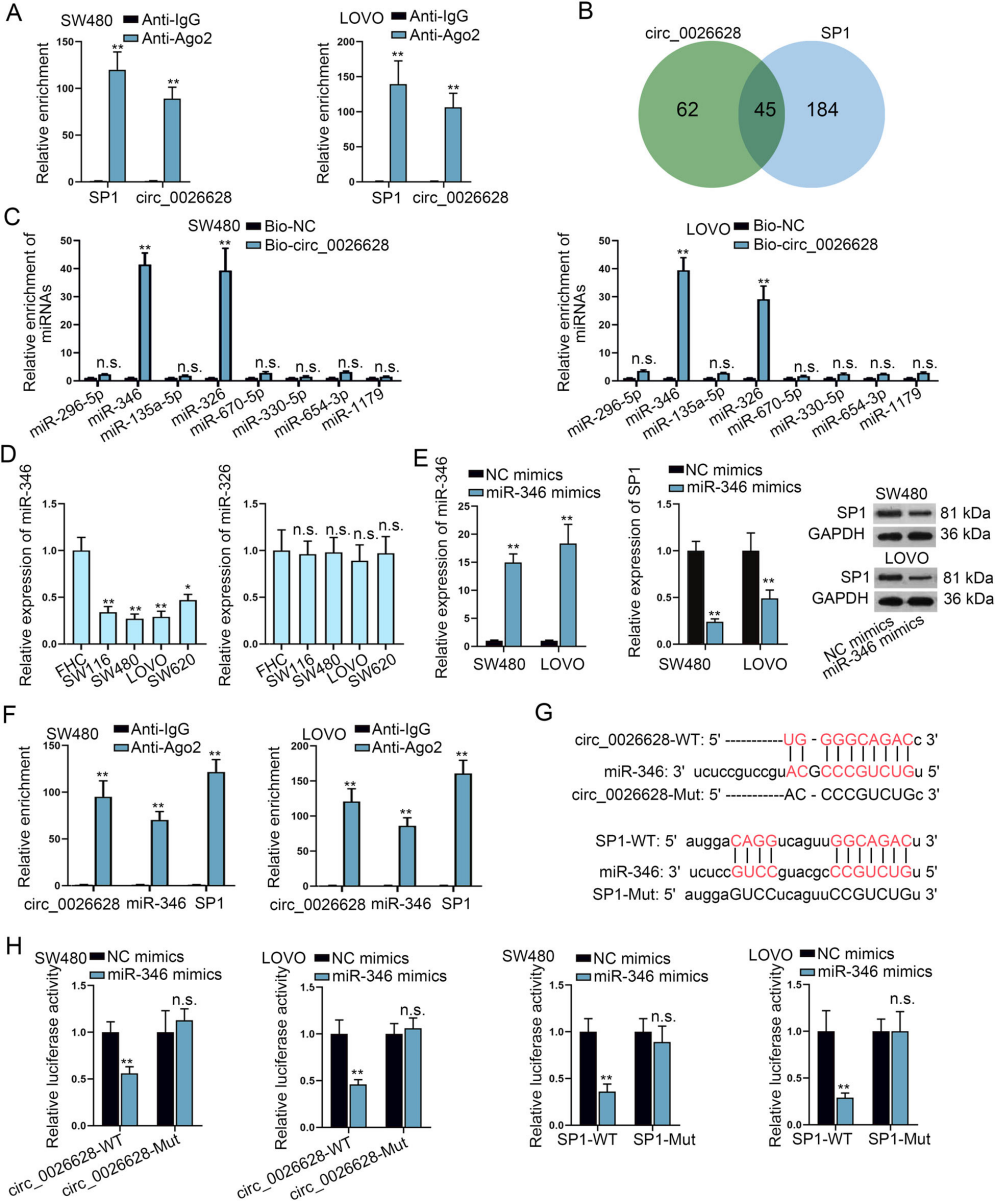
Circ_0026628 competed with SP1 for the binding of miR-346. **A** RIP assay revealed the enrichment of circ_0026628 and SP1 pulled down by anti-IgG and anti-Ago2. Student’s *t*-test. **B** Venn diagram revealed the miRNAs binding to both circ_0026628 and SP1 based on the starBase database. **C** RNA pull-down assay tested the enrichment of 8 candidate miRNAs pulled down by Bio-NC and Bio-circ_0026628-WT. Student’s *t*-test. **D** qRT-PCR analyzed the expression profile of miR-346 and miR-326 in CRC cells and the control cells. One-way ANOVA. **E** qRT-PCR verified the overexpression efficiency of miR-346; qRT-PCR and western blot disclosed the influence of miR-346 mimics on SP1 expression. Student’s *t*-test. **F** RIP assay estimated the enrichment of circ_0026628, miR-346, and SP1 in anti-Ago2 groups relative to anti-IgG groups. Student’s *t*-test. **G** Binding sites between circ_0026628/SP1 and miR-346 predicted by starBase, and the mutant circ_0026628/SP1 constructed based on indicated sites. **H** Luciferase reporter assay determined the luciferase activity of wild-type and mutant circ_0026628/SP1 in CRC cells with miR-346 mimics. Student’s *t*-test. *P < 0.05, **P < 0.01. “n.s.” indicates no significance.

Further, how circ_0026628 regulated SP1 in CRC was explored in the following assays. Since the previous assays revealed that circ_0026628 might exert post-transcriptional regulation in CRC cells, we wondered if circ_0026628 regulated SP1 via a ceRNA pattern. Seen from the data of the Ago2-RIP assay, we discovered that both circ_0026628 and SP1 were abundantly enriched in anti-Ago2 groups (Fig. 4A). Such phenomena indicated the existence of circ_0026628 and SP1 in RNA-induced silence complex (RISC), verifying the presence of a ceRNA network consist of them in CRC cells. On this basis, the common miRNAs binding with both circ_0026628 and SP1 were searched. According to the prediction results of starBase^20^, 229 miRNAs binding to SP1 (screening conditions: CLIP-Data ≥ 1, Degradome-Data ≥ 0, and pan-Cancer ≥ 4) and 107 miRNAs interacting with circ_0026628 (screening conditions: CLIP-Data ≥ 1 and Degradome-Data > 0) were discovered. Besides, the Venn diagram showed 45 miRNAs were shared by circ_0026628 and SP1 (Fig. 4B). Then, RNA pull-down assay results indicated that among the 45 miRNAs 8 were highly enriched by Bio-SP1 (Supplementary Fig. S2A), while only 2 of these 8 miRNAs (miR-346 and miR-326) were pulled down by Bio-circ_0026628 in CRC cells (Fig. 4C). Subsequently, the expression profile of miR-346 and miR-326 in CRC cells were examined. Results displayed that miR-346 was significantly downregulated in CRC cells compared with the control cells while miR-326 showed no distinct difference in expression between CRC cells and control cells (Fig. 4D). Then, we enhanced the expression of miR-346 and identified that upregulated miR-346 remarkably reduced the expression of SP1 at both mRNA and protein levels (Fig. 4E). Further, the RIP assay depicted that circ_0026628, miR-346, and SP1 were enriched in Ago2-mediated RISC (Fig. 4F). The binding sequences between circ_0026628/SP1 and miR-346 were predicted from starBase (Fig. 4G). We mutated the binding sequences for the following luciferase reporter assay. The results revealed that the luciferase activity of wild-type circ_0026628 or SP1 was remarkably reduced by miR-346 mimics while that of mutant circ_0026628 or SP1 was not impacted at the same time (Fig. 4H). Taken together, circ_0026628 competed with SP1 to bind with miR-346.

### Circ_0026628 partially depended on miR-346 to boost SP1

Then, we continued to investigate whether circ_0026628 functioned in CRC through a miR-346-mediated manner. Prior to that, we analyzed the role of miR-346 in CRC first. It was revealed that miR-346 upregulation hampered cell proliferation, migration, and EMT, and also weakened cell stemness in CRC (Supplementary Fig. S2B–G). These data certified miR-346 as a tumor-inhibitor in CRC. Further, the results of rescue assays revealed that inhibiting miR-346 partially rescued the effects of circ_0026628 depletion on cell proliferation, migration, EMT, and stemness (Supplementary Fig. S3A–G). More importantly, the reduction in SP1 expression owing to circ_0026628 deficiency was only partly recovered under miR-346 inhibition (Supplementary Fig. S3H). Combined with the previous data that upregulating SP1 perfectly rescued the effects of circ_0026628 silence on CRC cells, we speculated that circ_0026628 regulated SP1 through not only the miR-346-dependent pathway.

### Circ_0026628 recruited FUS to stabilize SP1

**Fig. 5:**
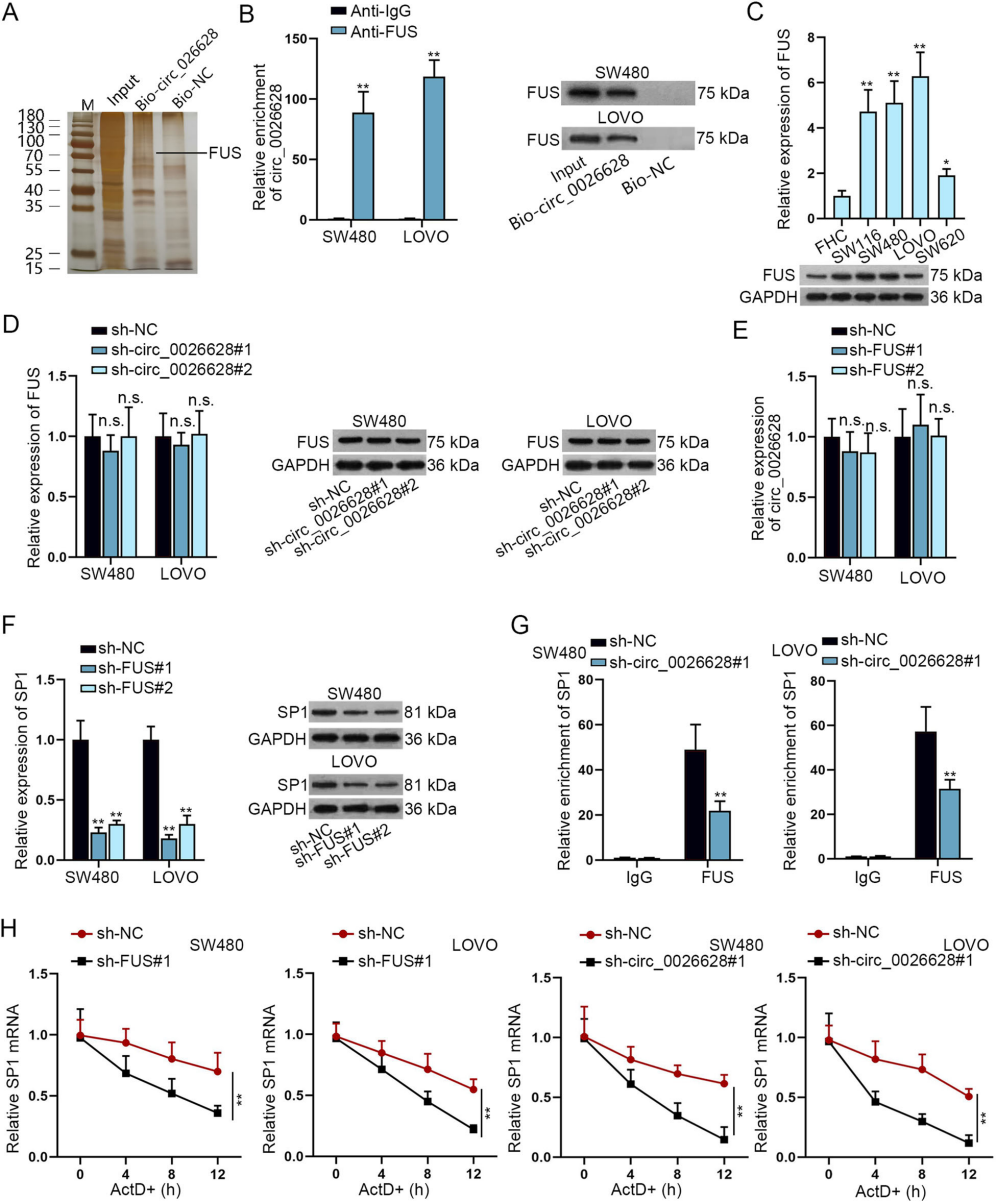
Circ_0026628 recruited FUS to stabilize SP1. **A** RNA pull-down followed by mass spectrometry validated FUS as the RBP to bind with circ_0026628. **B** RNA pull-down and RIP assays further confirmed the interaction betweencirc_0026628 and FUS in CRC cells. Student’s *t*-test. **C** qRT-PCR and western blot disclosed the expression profile of FUS in CRC cells and in control cells. One-way ANOVA. **D**. qRT-PCR and western blot examined the influence of circ_0026628 depletion on FUS expression. One-way ANOVA. **E** qRT-PCR revealed the influence of FUS deficiency on circ_0026628 expression. One-way ANOVA. **F** qRT-PCR and western blot revealed the influence of silenced FUS on SP1 expression. One-way ANOVA. **G** RIP assay tested the enrichment of SP1 precipitated by anti-IgG and anti-FUS when circ_0026628 was silenced or not. Two-way ANOVA. **H** qRT-PCR detected SP1 mRNA level under ActD treatment in CRC cells with or without circ_0026628 or FUS knockdown. Two-way ANOVA. ^*^P < 0.05, **P < 0.01. “n.s.” indicates no significance.

Subsequently, we aimed at disclosing another way whereby circ_0026628 regulated SP1 in CRC. Since circRNAs can also recruit specific RBPs to play post-transcriptional regulatory roles, we then conducted an RNA pull-down assay to uncover the potential RBP for circ_0026628. Based on mass spectrometry analysis of proteins, which were pulled down by Bio-circ_0026628 rather than the control group, FUS was identified (Fig. 5A). Additionally, the interaction between FUS and circ_0026628 was further validated in SW480 and LOVO cells by RNA pull-down and RIP assays (Fig. 5B). Consistently, the data from IF and FISH assays also proved the co-localization of circ_0026628 and FUS mainly in the cytoplasm of these two CRC cells (Supplementary Fig. S4A). Hence, we confirmed FUS as the RBP interacting with circ_0026628 in CRC. Then, we identified that FUS was significantly upregulated in CRC cells relative to the control cells (Fig. 5C). Moreover, silencing circ_0026628 had no influence on FUS mRNA and protein levels (Fig. 5D). In the meantime, we knocked down FUS expression by sh-FUS#1/2 (Supplementary Fig. S4B), and figured out that downregulated FUS had no impacts on circ_0026628 expression while obviously reduced the expression of SP1 (Fig. 5E, F). Importantly, FUS is the potential to bind SP1 based on the starBase database. Furthermore, the RIP assay disclosed that SP1 was significantly pulled down by anti-FUS, while circ_0026628 knockdown markedly reduced the enrichment of SP1 in an anti-FUS group (Fig. 5G). In other words, SP1 mRNA could interact with FUS, and the interaction between them was hampered under the absence of circ_0026628. Of note, we discovered that overexpressing FUS did not affect the relative enrichment of SP1 mRNA in Bio-miR-346 groups (Supplementary Fig. S4C, D), proofing that the interaction of FUS with SP1 mRNA had no influence on the binding of miR-346 to SP1 3′UTR. Thereafter, we tested the impact of FUS and circ_0026628 on SP1 through treating CRC cells with ActD. The results demonstrated that when FUS or circ_0026628 was knocked down, SP1 mRNA level was reduced faster under ActD treatment (Fig. 5H), suggesting that FUS/circ_0026628 depletion lowered the stability of SP1 mRNA. To sum up, circ_0026628 interacted with FUS protein to stabilize SP1 in CRC cells.

### Circ_0026628-mediated effects on CRC cells via Wnt/β-catenin signaling pathway

**Fig. 6:**
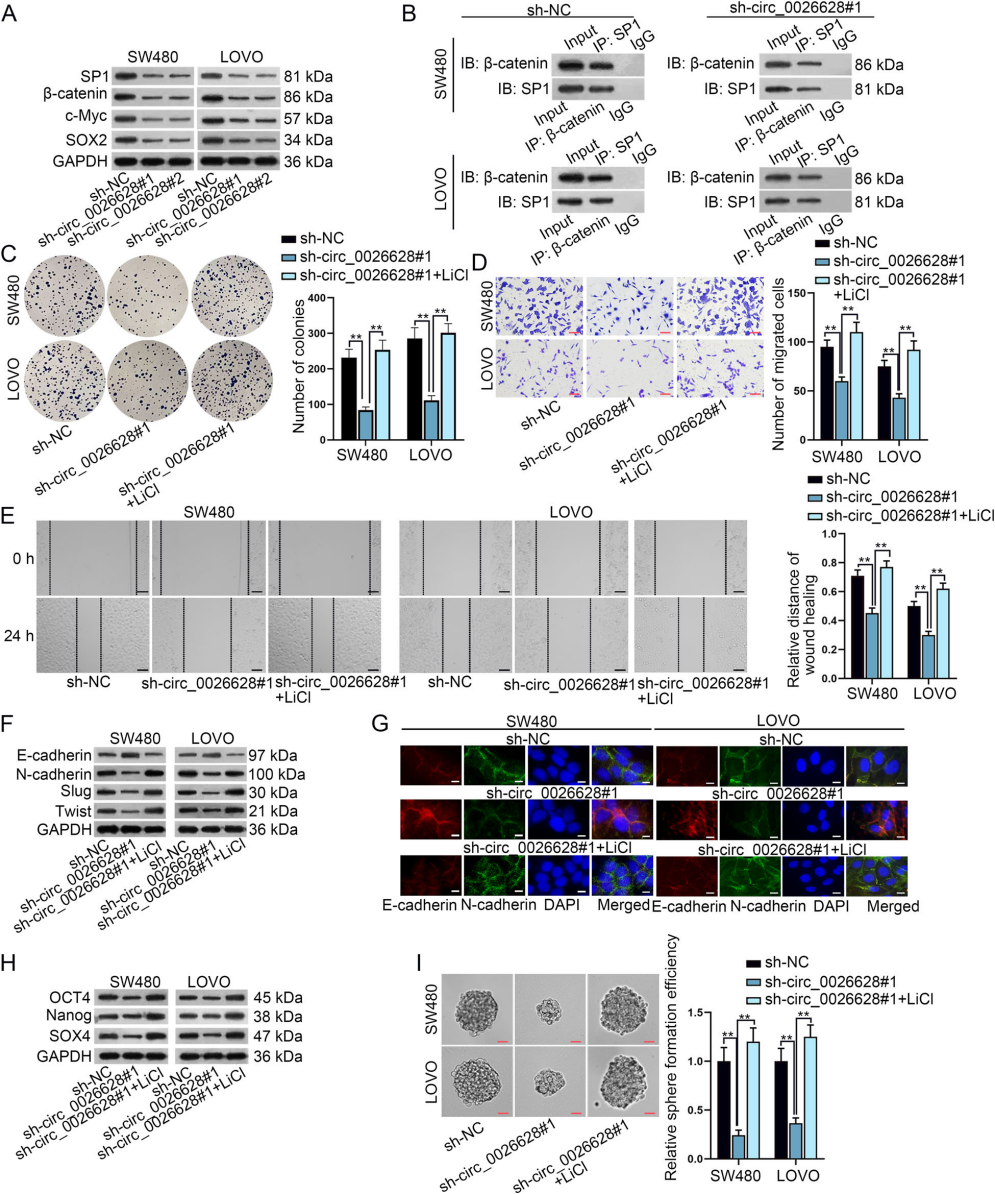
Circ_0026628 mediated CRC cells via Wnt/β-catenin signaling pathway. **A** Western blot determined the protein levels of SP1,β-catenin, c-Myc, and SOX2 in CRC cells when circ_0026628 was silenced or not. **B** CoIP assay plus western blot detected the changes in the interaction between SP1 and β-catenin when circ_0026628 was silenced. **C**–**E** Colony formation, transwell (scale bar: 180 μm) and wound-healing assays (scale bar: 120 μm) evaluated the rescue effects of LiCl treatment on the proliferation and migration of circ_0026628-silenced CRC cells. One-way ANOVA. **F**, **G** Western blotting and IF staining assay (scale bar: 150 μm) revealed the rescue effects of LiCl on the EMT process in circ_0026628-silenced CRC cells. **H**, **I** Western blot and sphere formation assay (scale bar: 150 μm) explored the effects of LiCl on circ_0026628 silence-inhibited cell stemness. One-way ANOVA. **P < 0.01.

Since SP1 could activate Wnt/β-catenin signaling, here we examined whether circ_0026628 could trigger Wnt/β-catenin signaling via targeting SP1. In this case, the expression of the core factor (β-catenin) and two well-recognized downstream targets (c-Myc and SOX2) of this pathway, as well as that of SP1, was detected in circ_0026628-silenced CRC cells via western blot. It was revealed that all the levels of SP1,β-catenin, c-Myc, and SOX2 were noticeably reduced by silenced circ_0026628 (Fig. 6A). A previous study has revealed that SP1 interacted with β-catenin to accumulate β-catenin and promoted its nuclear translocation, thus activating Wnt/β-catenin signaling^22^. In the present study, we wondered whether circ_0026628 influenced the interaction between β-catenin and SP1 to affect this pathway. As expected, the level of nuclear β-catenin was apparently lessened and the translocation of β-catenin into the nucleus was also hindered in face of circ_0026628 deficiency (Supplementary Fig. S4E). Significantly, all these phenomena were attributed to the declined levels of both SP1 and β-catenin and the blocked interaction between them in circ_0026628-silenced CRC cells (Fig. 6B and Supplementary Fig. S4F). Further, LiCl, the agonist of Wnt/β-catenin pathway^23^, was employed for following rescue assays. Results depicted that LiCl treatment completely rescued the suppressive effects of silenced circ_0026628 on CRC cell proliferation, migration, EMT, and stemness (Fig. 6C–I). All in all, we concluded that circ_0026628 mediated biological functions in CRC cells via upregulating SP1 to stimulate the Wnt/β-catenin pathway.

### SOX2 transcriptionally activated SP1 to elevate circ_0026628 expression

**Fig. 7:**
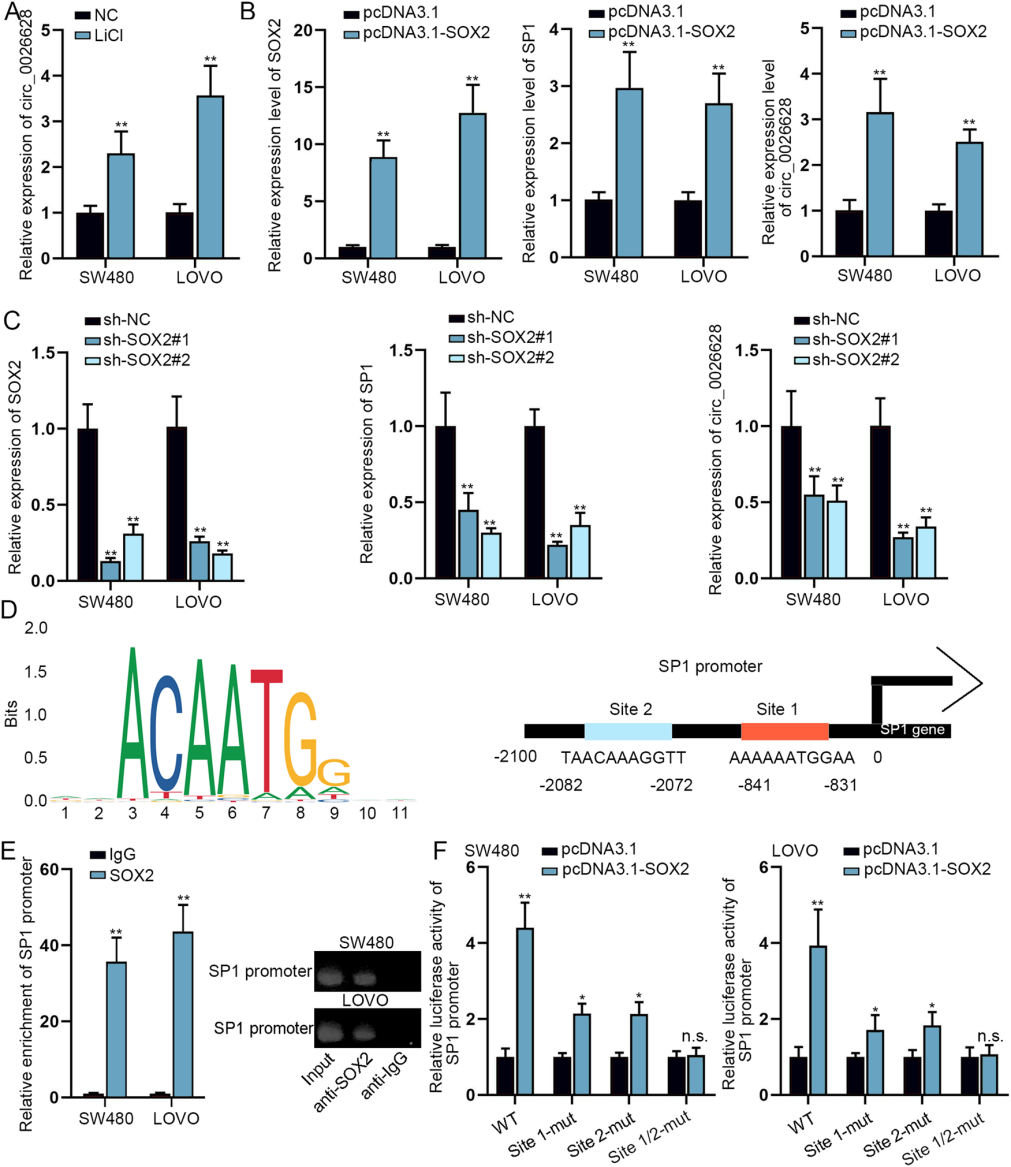
SOX2 activated SP1 transcription to enhance circ_0026628. **A** qRT-PCR revealed the influence of LiCl on circ_0026628 expression. Student’s *t*-test. **B** qRT-PCR verified the overexpression efficiency of SOX2 and the influence of upregulated SOX2 on SP1 and circ_0026628 expression. Student’s *t*-test. **C** qRT-PCR verified the knockdown efficiency of SOX2 and the influence of downregulated SOX2 on SP1 and circ_0026628 expression. One-way ANOVA. **D** JASPAR predicted the DNA-binding Motif of SOX2 and 2 binding sites of SOX2 on SP1 promoter. **E** ChIP assay revealed the enrichment of SP1 promoter in anti-SOX2 group relative to anti-IgG group. Student’s *t*-test. **F** Luciferase reporter assay unveiled the luciferase activity of reporters with wild-type or mutant SP1 promoter. Student’s *t*-test. *P < 0.05, **P < 0.01. “n.s.” indicates no significance.

Moreover, we used LiCl to treat CRC cells and figured out that circ_0026628 expression was enhanced by LiCl treatment (Fig. 7A). Since SOX2 was the downstream target genes of the Wnt/β-catenin pathway and SOX2 commonly served as the transcription factor, we wondered if SOX2 could transcriptionally activate SP1 to positively regulate circ_0026628. The expression of SOX2 was enhanced by pcDNA3.1-SOX2 in CRC cells, leading to elevated expression of SP1 and circ_0026628 (Fig. 7B). In contrast, the downregulation of SOX2 resulted in a significant decrease in the levels of SP1 and circ_0026628 (Fig. 7C). These results suggested that SOX2 positively modulate SP1 and circ_0026628 expression in CRC. Based on JASPAR (http://jaspar.genereg.net/), the DNA motif of SOX2 and two potential binding sequences of SOX2 in SP1 promoter (2100 bp sequence upstream of SP1 gene sequence, genomic position: chr12:53771879-53773978) were illustrated in Fig. 7D. We conducted a ChIP assay and identified that the SP1 promoter was remarkably pulled down by anti-SOX2 (Fig. 7E). Moreover, a luciferase reporter assay was implemented. Results manifested that the luciferase activity of wild-type SP1 promoter was significantly enhanced by SOX2 overexpression, while such enhancement effect was a bit mitigated when site 1 or 2 was mutated but completely abrogated when both sites 1 and 2 were mutated (Fig. 7F). Thus, we concluded that SOX2 bound to both sites in SP1 promoter, finally upregulating circ_0026628 in a transcriptional way.

On the whole, circ_0026628 sponged miR-346 and recruited FUS protein to elevate SP1, therefore triggering the downstream Wnt/β-catenin pathway to accelerate malignancy in CRC. In return, SOX2, the downstream gene of the Wnt/β-catenin pathway, further transcriptionally activated SP1 to enhance circ_0026628.

## Discussion

With the advancement of bioinformatics approaches and deep sequencing, comprehensive studies of circRNAs are allowed in human diseases, including cancers^24–26^. CircRNAs like circ_0005963^10^, circ_0005100^11^, and circ_0009361^12^ are known as putative biomarkers for CRC. However, the functions of a large number of circRNAs in CRC have not been researched yet. The present study detected the differentially expressed circRNAs which shared the same pre-mRNA with SP1, an oncogene with elevated expression in CRC. Consequently, circ_0026628 was identified as the only circRNA that was upregulated in all four kinds of CRC cells. Moreover, loss-of-function assay results showed that silencing circ_0026628 weakened cell proliferation, migration, EMT, and stemness in CRC. These findings also suggested circ_0026628 as a promising biomarker for CRC. Since the forming process of exonic circRNAs could affect alternative splicing of their relevant pre-mRNAs, which potentially leads to altered host gene expression^27^, we detected the influence of circ_0026628 on SP1 expression. The results revealed that circ_0026628 positively regulated SP1 expression. Importantly, we testified that circ_0026628 facilitated CRC cell proliferation, migration, EMT, and stemness via upregulating SP1.

In the present work, we uncovered the cytoplasmic localization of most circ_0026628 in CRC cells, which established the potential that circ_0026628 played a post-transcriptional regulatory role in CRC. For most circRNAs, two mechanisms have been reported as the most prevalent manner to exert post-transcriptional regulations on protein-coding genes^28^. One is the so-called ceRNA mechanism, in which circRNAs serve as miRNA sponges to protect mRNAs from miRNAs-induced silencing^29^. Since we identified that circ_0026628 and SP1 were abundantly enriched in RISC, we speculated that circ_0026628 could regulate SP1 via the ceRNA pattern. By bioinformatics analysis and a series of mechanistic assays, miR-346 was found to bind with both circ_0026628 and SP1. Besides, we attested that miR-346 was lowly-expressed in CRC cells, consistent with former findings that miR-346 was downregulated in CRC^30^. Further, we certified that circ_0026628 served as a ceRNA against miR-346 to elevate SP1 expression. Nowadays, the mediating role of miR-346 in the ceRNA pattern has been widely validated. MIR17HG serves as ceRNA against miR-346/miR-425-5p to up-regulate TAL1 in glioma cells^31^. CircFBLIM1 functions as a miR-346 sponge to regulate FBLIM1 expression, thus promoting hepatocellular cancer progression^32^. LncRNA DGCR5 represses hepatocellular carcinoma cell growth, migration, and invasion through sponging miR-346 to elevate KLF14 expression^33^. Apart from the present study, modulation of circRNAs on their linear mRNAs via the ceRNA pattern has also been reported in many studies. He et al.^34^ revealed that circGFRA1 and GFRA1 function as ceRNAs by binding with miR-34a to promote triple-negative breast cancer development. CircRNA-ENO1 elevates the expression of its host gene ENO1 to promote glycolysis and tumor progression in lung adenocarcinoma through sequestering miR-22-3p^35^.

After we have verified the ceRNA mechanism, we found that inhibiting miR-346 partially rescued the effects of silenced circ_0026628, indicating that circ_0026628 might regulate linear SP1 via another pattern. Hence, we then focused on another common function for circRNAs to interact with RBPs to modulate gene expression at post-transcriptional level^36^. For example, circPABPN1 regulates the linear PABPN1 through binding to HuR in human cervical carcinoma cells^37^. Thus, we wondered if circ_0026628 recruited specific RBP to post-transcriptional regulate SP1. After the mechanistic assays, we verified that circ_0026628 bound to FUS protein mainly in the cytoplasm of CRC cells to facilitate the stabilization of SP1. Previously, studies have suggested that FUS resides in the nucleus of different cell types to affect gene transcription. As an example, Dhar et al.^38^ have pointed out that FUS binds to MnSOD promoter to MnSOD transcription. Nevertheless, there are also numerous evidences supporting the cytoplasmic role of FUS. For instance, Udagawa et al.^39^ discovered that FUS also exists in the cytoplasm and the cytoplasmic FUS interacts with GluA1 Mrna to enhance its stability. Notably, the recruiting role of circRNAs in strengthening FUS-induced stabilization on mRNAs in the cytoplasm has also been reported previously. Di et al.^40^ uncovered that SNHG14 elevates EZH2 expression via interacting with FUS to stabilizing EZH2 mRNA in CRC cells.

An increasing number of studies have uncovered that SP1 is involved in the activation of the Wnt/β-catenin pathway^16,41,42^. Moreover, Mir et al.^22^ have disclosed that SP1 directly interacts with β-catenin and promoted the nuclear translocation of β-catenin. Consistently, we demonstrated that circ_0026628 promoted CRC cell proliferation, migration, EMT, and stemness via activating the Wnt/β-catenin pathway through fortifying the interaction between SP1 and β-catenin. Moreover, we found out that circ_0026628 expression was upregulated after activating the Wnt/β-catenin pathway. Thus, we speculated that the downstream gene of the Wnt/β-catenin pathway could regulate circ_0026628 in turn. Herein, it was revealed that SOX2 was exactly the transcriptional activator for SP1, which finally elevated the level of SP1 mRNA-derived circ_0026628. SOX2 is a common stemness biomarker, which also functions as a transcription factor. For example, Lu et al.^43^ concluded that miR-200c targets Sox2 to inhibit CRC cell stemness, growth, and migration. Liu et al.^44^ revealed that SOX2 binds to lncRNA HBL1 promoter to activate HBL1 transcription.

Conclusively, the present study firstly demonstrated that circ_0026628 positively modulated its cognate gene of SP1 to activate the Wnt/β-catenin pathway via sponging miR-346 and recruiting FUS. Besides, the downstream target of the Wnt/β-catenin pathway, SOX2, further transcriptionally activated SP1 to induce circ_0026628 upregulation. Further, we testified that such feedback mechanism facilitated CRC cell proliferation, migration, EMT, and stemness. Significantly, our work supported circ_0026628 is a promising therapeutic target for CRC. Nonetheless, the lack of clinical data are the main limitation of the current study, which will be solved in the future.

## Supplementary information

Supplementary figure legends

Figure S1.

Figure S2.

Figure S3.

Figure S4.

Supplementary table 1

Supplementary file 1

## References

[1] Bray F (2018). Global cancer statistics 2018: GLOBOCAN estimates of incidence and mortality worldwide for 36 cancers in 185 countries. CA Cancer J. Clin..

[2] Pan Z (2019). Hsa_circ_0006948 enhances cancer progression and epithelial-mesenchymal transition through the miR-490-3p/HMGA2 axis in esophageal squamous cell carcinoma. Aging.

[3] Chen J (2019). circPTN sponges miR-145-5p/miR-330-5p to promote proliferation and stemness in glioma. J. Exp. Clin. Cancer Res..

[4] Fan X (2015). Single-cell RNA-seq transcriptome analysis of linear and circular RNAs in mouse preimplantation embryos. Genome Biol..

[5] Granados-Riveron JT, Aquino-Jarquin G (2016). The complexity of the translation ability of circRNAs. Biochim. Biophys. Acta.

[6] Barrett SP, Salzman J (2016). Circular RNAs: analysis, expression and potential functions. Development.

[7] Memczak S (2013). Circular RNAs are a large class of animal RNAs with regulatory potency. Nature.

[8] Guo JU, Agarwal V, Guo H, Bartel DP (2014). Expanded identification and characterization of mammalian circular RNAs. Genome Biol..

[9] Li Z (2015). Exon-intron circular RNAs regulate transcription in the nucleus. Nat. Struct. Mol. Biol..

[10] Wang, X. et al. Exosome-delivered circRNA promotes glycolysis to induce chemoresistance through the miR-122-PKM2 axis in colorectal cancer. *Mol. Oncol.* **14**, 539–555 (2020). 10.1002/1878-0261.12629 PMC7053238 31901148

[11] Li Y (2019). A novel circFMN2 promotes tumor proliferation in CRC by regulating the miR-1182/hTERT signaling pathways. Clin. Sci..

[12] Geng Y (2019). Hsa_circ_0009361 acts as the sponge of miR-582 to suppress colorectal cancer progression by regulating APC2 expression. Clin. Sci..

[13] Du WW (2016). Foxo3 circular RNA retards cell cycle progression via forming ternary complexes with p21 and CDK2. Nucleic Acids Res..

[14] Zhu YJ (2019). Circular RNAs negatively regulate cancer stem cells by physically binding FMRP against CCAR1 complex in hepatocellular carcinoma. Theranostics.

[15] Chen, X. et al. METTL14 suppresses CRC progression via regulating N6-methyladenosine-dependent primary miR-375 processing. *Mol. Ther.* **28**, 599–612 (2020). 10.1016/j.ymthe.2019.11.016 PMC7001002 31839484

[16] Liu B (2019). MiR-29b/Sp1/FUT4 axis modulates the malignancy of leukemia stem cells by regulating fucosylation via Wnt/beta-catenin pathway in acute myeloid leukemia. J. Exp. Clin. Cancer Res..

[17] Zhan T, Rindtorff N, Boutros M (2017). Wnt signaling in cancer. Oncogene.

[18] Jiang S (2018). Ubiquitin-specific peptidase 22 contributes to colorectal cancer stemness and chemoresistance via Wnt/beta-catenin pathway. Cell. Physiol. Biochem..

[19] Gu C (2019). MiR-532-3p suppresses colorectal cancer progression by disrupting the ETS1/TGM2 axis-mediated Wnt/beta-catenin signaling. Cell Death Dis..

[20] Li JH, Liu S, Zhou H, Qu LH, Yang JH (2014). starBase v2.0: decoding miRNA-ceRNA, miRNA-ncRNA and protein-RNA interaction networks from large-scale CLIP-Seq data. Nucleic Acids Res..

[21] Glazar P, Papavasileiou P, Rajewsky N (2014). circBase: a database for circular RNAs. RNA.

[22] Mir, R., Sharma, A., Pradhan, S. J. & Galande, S. Regulation of transcription factor SP1 by the beta-catenin destruction complex modulates Wnt response. *Mol. Cell. Biol.* **38**, e00188-18 (2018). 10.1128/MCB.00188-18 PMC6206460 30181396

[23] Li J, Zhang Y, Zhao Q, Wang J, He X (2015). MicroRNA-10a influences osteoblast differentiation and angiogenesis by regulating beta-catenin expression. Cell. Physiol. Biochem..

[24] Chen B (2018). circEPSTI1 as a prognostic marker and mediator of triple-negative breast cancer progression. Theranostics.

[25] Wang R (2018). CircNT5E acts as a sponge of miR-422a to promote glioblastoma tumorigenesis. Cancer Res..

[26] Han D (2017). Circular RNA circMTO1 acts as the sponge of microRNA-9 to suppress hepatocellular carcinoma progression. Hepatology.

[27] Liu J, Liu T, Wang X, He A (2017). Circles reshaping the RNA world: from waste to treasure. Mol. Cancer.

[28] He J, Xie Q, Xu H, Li J, Li Y (2017). Circular RNAs and cancer. Cancer Lett..

[29] Hansen TB (2013). Natural RNA circles function as efficient microRNA sponges. Nature.

[30] Liang Y (2020). Hsa_circ_0026416 promotes proliferation and migration in colorectal cancer via miR-346/NFIB axis. Cancer Cell Int..

[31] Cao S (2019). FXR1 promotes the malignant biological behavior of glioma cells via stabilizing MIR17HG. J. Exp. Clin. Cancer Res..

[32] Bai N (2018). circFBLIM1 act as a ceRNA to promote hepatocellular cancer progression by sponging miR-346. J. Exp. Clin. Cancer Res..

[33] Wang YG, Liu J, Shi M, Chen FX (2018). LncRNA DGCR5 represses the development of hepatocellular carcinoma by targeting the miR-346/KLF14 axis. J. Cell. Physiol..

[34] He R (2017). circGFRA1 and GFRA1 act as ceRNAs in triple negative breast cancer by regulating miR-34a. J. Exp. Clin. Cancer Res..

[35] Zhou J (2019). CircRNA-ENO1 promoted glycolysis and tumor progression in lung adenocarcinoma through upregulating its host gene ENO1. Cell Death Dis..

[36] Huang A, Zheng H, Wu Z, Chen M, Huang Y (2020). Circular RNA-protein interactions: functions, mechanisms, and identification. Theranostics.

[37] Abdelmohsen K (2017). Identification of HuR target circular RNAs uncovers suppression of PABPN1 translation by CircPABPN1. RNA Biol..

[38] Dhar SK (2014). FUsed in sarcoma is a novel regulator of manganese superoxide dismutase gene transcription. Antioxid. Redox Signal..

[39] Udagawa T (2015). FUS regulates AMPA receptor function and FTLD/ALS-associated behaviour via GluA1 mRNA stabilization. Nat. Commun..

[40] Di W (2019). Long noncoding RNA SNHG14 facilitates colorectal cancer metastasis through targeting EZH2-regulated EPHA7. Cell Death Dis..

[41] Monteleone, E. et al. SP1 and STAT3 functionally synergize to induce the RhoU small GTPase and a subclass of non-canonical WNT responsive genes correlating with poor prognosis in breast cancer. *Cancers*. **11**, 101 (2019). 10.3390/cancers11010101 PMC6356433 30654518

[42] Kim MK (2009). An integrated genome screen identifies the Wnt signaling pathway as a major target of WT1. Proc. Natl Acad. Sci. USA.

[43] Lu YX (2014). Regulation of colorectal carcinoma stemness, growth, and metastasis by an miR-200c-Sox2-negative feedback loop mechanism. Clin. Cancer Res..

[44] Liu J, Li Y, Lin B, Sheng Y, Yang L (2017). HBL1 is a human long noncoding RNA that modulates cardiomyocyte development from pluripotent stem cells by counteracting MIR1. Dev. Cell.

