# Supplementary material for: Hsa_circ_0026628 promotes the development of colorectal cancer by targeting SP1 to activate the Wnt/β-catenin pathway
Source: Cell Death Dis. 2021 Aug 21;12(9):802. doi: 10.1038/s41419-021-03794-6 (PMC8380248; doi:10.1038/s41419-021-03794-6)
Supplement: Supplementary file 1 — Supplementary figure legends [file 41419_2021_3794_MOESM1_ESM.docx]

**Supplementary figure legends**

**Figure S1.**

**A.** qRT-PCR verified the depletion efficiency of circ_0026628. One-way ANOVA.

**B.** qRT-PCR tested the impact of circ_0026628 knockdown on the level of SP1 pre-mRNA. One-way ANOVA.

**C.** CCK-8 detected the proliferation of CRC cells with or without silenced circ_0026628. Two-way ANOVA.

**D.** The images of spheres after 3 and 10 days of incubation in CRC cells with or without circ_0026628 silencing.

**E.** qRT-PCR verified the overexpression efficiency of circ_0026628 and the influence of up-regulated circ_0026628 on SP1 expression. Student’s t-test.

**F.** qRT-PCR verified the overexpression efficiency of SP1. Student’s t-test.

**G.** CCK-8 detected the proliferation of CRC cells under different conditions. Two-way ANOVA. ^**^P<0.01.

**Figure S2.**

**A.** RNA pull down assay analyzed the interaction of SP1 with 45 miRNAs in CRC cells. Student’s t-test.

**B.** CCK-8 detected the proliferation of CRC cells transfected with NC mimics or miR-346 mimics. Two-way ANOVA.

**C-G.** Colony formation assay (C), transwell assay (D), wound healing assay (E), western blotting of EMT biomarker (F) and sphere formation assay (G) examined the effects of miR-346 upregulation on CRC cell functions. Student’s t-test. ^**^P<0.01.

**Figure S3.**

**A.** CCK-8 detected the proliferation of CRC cells under circ_0026628 silencing or together with miR-346 inhibition. Two-way ANOVA.

**B-G.** Colony formation assay (B), transwell assay (C), wound healing assay (D), western blotting of EMT biomarker (E) and stemness biomarkers (F), sphere formation assay (G) revealed the rescue effects of miR-346 inhibition on circ_0026628-silenced CRC cells. One-way ANOVA.

**H.** qRT-PCR analyzed SP1 level in CRC cells under above contexts. One-way ANOVA. ^*^P<0.05, ^**^P<0.01.

**Figure S4.**

**A.** FISH assay showed circ_0026628 staining and IF assay detected FUS staining.

**B.** qRT-PCR and western blot tested the depletion efficiency of FUS. One-way ANOVA.

**C.** qRT-PCR verified the overexpression efficiency of FUS in both CRC cells. Student’s t-test.

**D.** RNA pull down analyzed the influence of FUS overexpression on the interaction between miR-346 and SP1 mRNA. Student’s t-test.

**E.** Western blot detected the level of β-catenin in cytoplasmic and nuclear fractions of CRC cells with or without silenced circ_0026628.

**F.** IF staining showed the co-localization of SP1 and β-catenin in CRC cells when circ_0026628 was silenced or not. ^**^P<0.01.

**Supplementary table 1.** The sequences of primers used for qRT-PCR.

**Supplementary file 1.** The sequence of SP1 promoter.
