# Supplementary figures and images for: Hsa_circ_0026628 promotes the development of colorectal cancer by targeting SP1 to activate the Wnt/β-catenin pathway
Source: Cell Death Dis. 2021 Aug 21;12(9):802. doi: 10.1038/s41419-021-03794-6 (PMC8380248; doi:10.1038/s41419-021-03794-6)

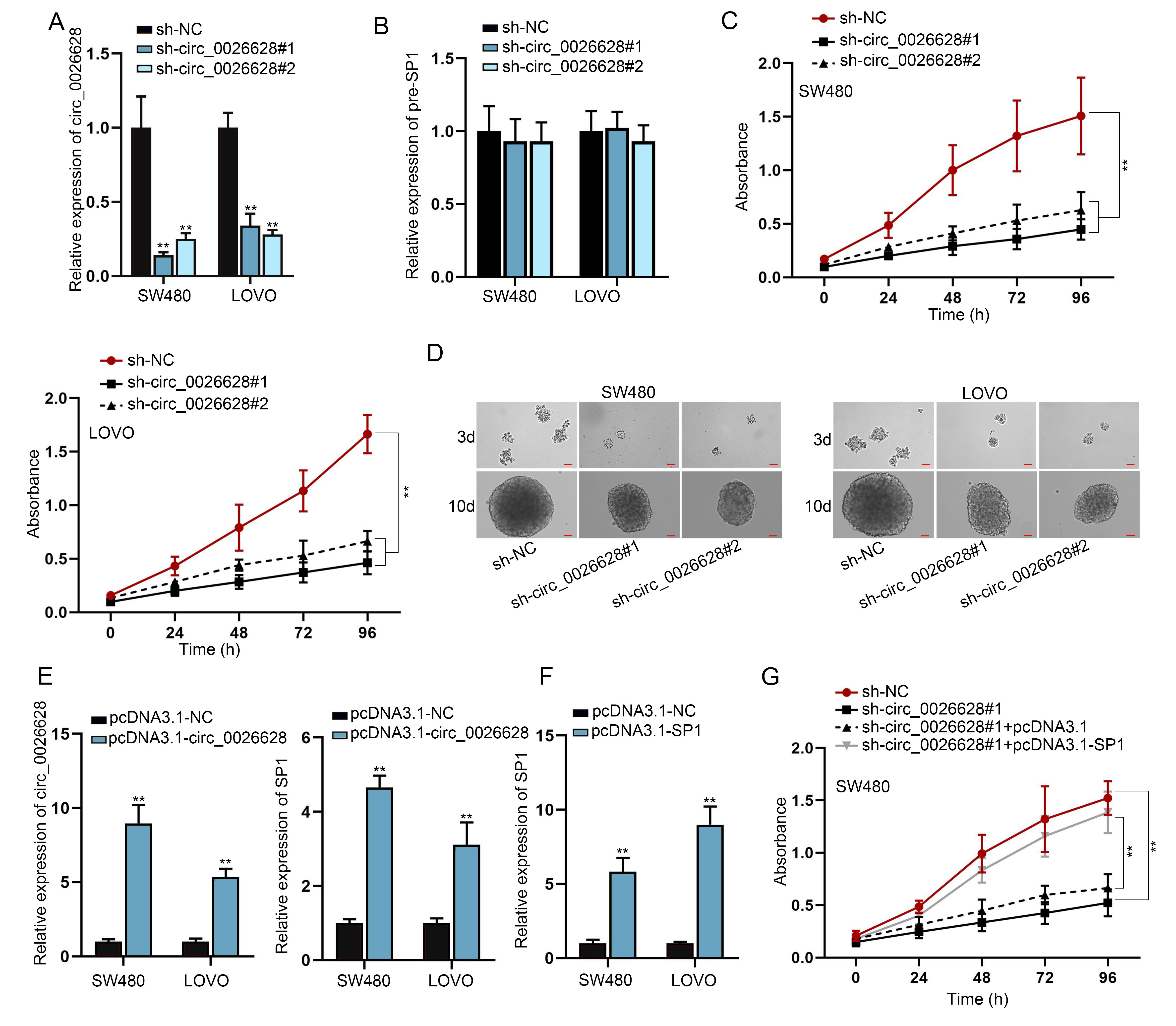

Supplement: Supplementary file 2 — Figure S1. [file 41419_2021_3794_MOESM2_ESM.tif]

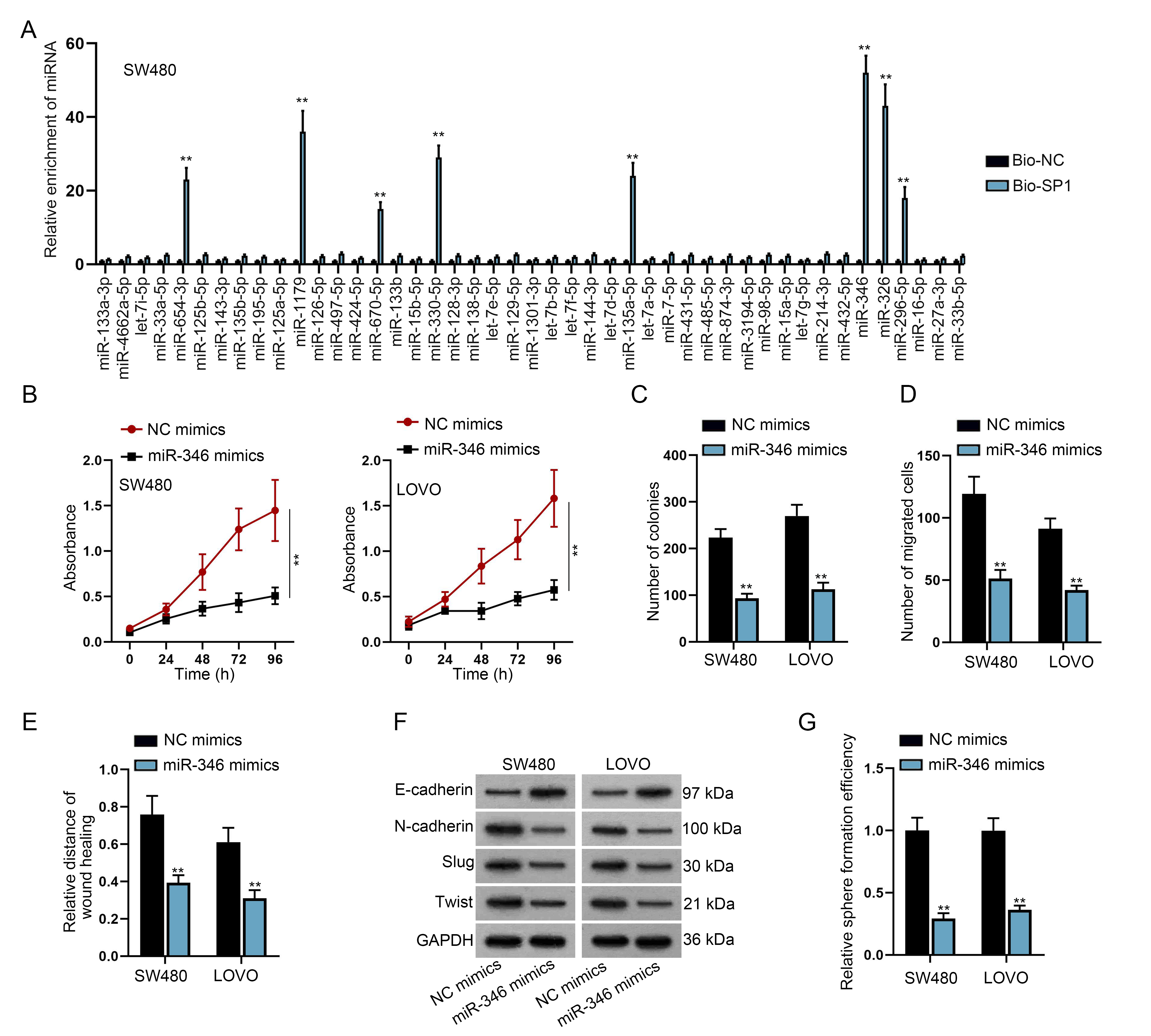

Supplement: Supplementary file 3 — Figure S2. [file 41419_2021_3794_MOESM3_ESM.tif]

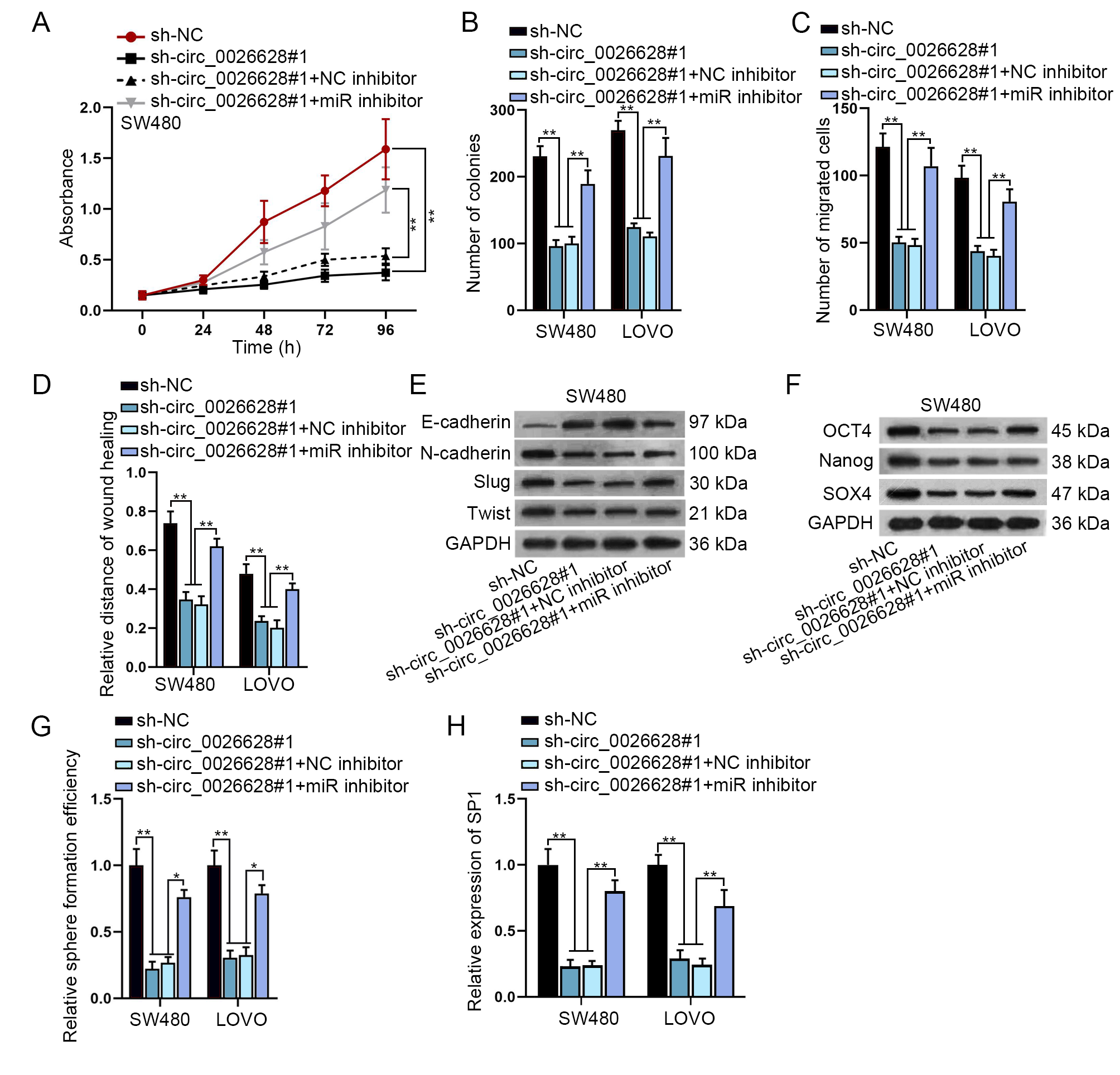

Supplement: Supplementary file 4 — Figure S3. [file 41419_2021_3794_MOESM4_ESM.tif]

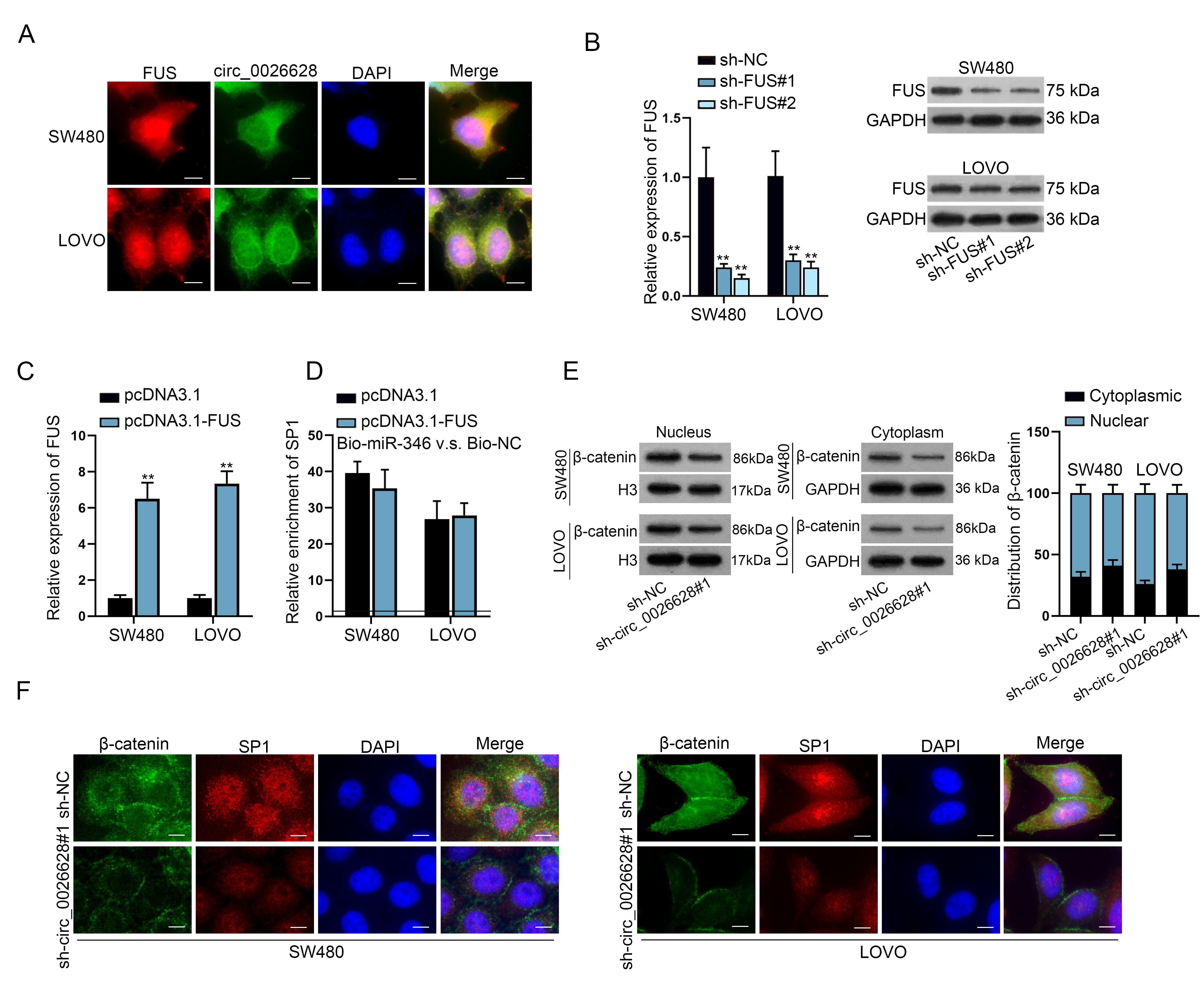

Supplement: Supplementary file 5 — Figure S4. [file 41419_2021_3794_MOESM5_ESM.tif]
