# Supplementary material for: Hsa_circ_0026628 promotes the development of colorectal cancer by targeting SP1 to activate the Wnt/β-catenin pathway
Source: Cell Death Dis. 2021 Aug 21;12(9):802. doi: 10.1038/s41419-021-03794-6 (PMC8380248; doi:10.1038/s41419-021-03794-6)
Supplement: Supplementary file 7 — Supplementary file 1 [file 41419_2021_3794_MOESM7_ESM.docx]

>hg19_knownGene_uc001scw.3 range=chr12:53771879-53773978 5'pad=0 3'pad=0 strand=+ repeatMasking=none

CTGACCTCAGGTGATCCGCCTGCCTCGGCCTCCCAAAATGCTGGGATTAC

AGGCATGAGCCACCGCGCCCAGTCTATATATTTACTTATTTTGGAGCAAG

GTCTTGCTTTGTTGTCCTGGCTGGAGTGCAGTGGCAGGATTTGAGCTCAC

TGCAGCCTCAACCTCCTGGGTTCAAGCGATTCTTCCACCCCAGCCTCCCG

AGTAGCTGGGACTACTGGCGCACATCACCACGTCTGGCTAATTTTTGTTA

TTTTTTGTGGAGACGGGGTTTCACCATGTTGCCCAGGCTGGTCTTTAACT

CCTGGGCTCAAGCGATCCGCCCGCCTCGGCCTCCCATAGTGCTGGGATTA

CAGGTCTGAGCCATTGCGCCCGGCGGCAGTTTAATTCCCTCAAGAATTTA

TAGAAAACTTTTGGTGGAACTATTCTGGGTTGGAATCTAACACTACAACT

CATTTGCTTTGTGACCTTGGGTAAGTCACTTCTCAGCCTAAATGTCTTCA

CTTATAAAATACATAATATAGCTGATACTTAATAAAATCTTCCTTTTAAG

GATGACATGGAATACTAACTGCCTCCCTGACTTAGGCACCTAACACGGTA

GGCAGTCAGCAATCACTTCACCTTTTTACCTGTTTAAAGCAGCAGAGGCC

TCAGTTCTTACCTTCAAAGGGCTTAAAATAGTTTAGGAGAATTCACATTT

TAAAACATAAGAACATATAAGCAGGTGACAACATATAAGATACAAATACT

GAAGGACATGACCTCATCGTGTCATTGACAAGCTATTAAGGCCGGCTCCA

CCAAAACACGGATAAAGAGGAGGCCAGAAATCCAGGTGCCTGCAGTAAAG

TTTCTTAACCTTCGTGATTGCAAAAAGCTGGAGCTCAGCTATCTTGCTTT

ATGCATAGGCGGTATTTACTATTAAGGGGGAAAAAATGGAAGTGACTTAT

CCGCACTTTAGCCTCAAGGCTTGAAAAGTTAACCAGTCGTTTAAGTGGTT

AGCGCCTTTGTCTGGGGGAACTTAATAAAATCGCGTTTTCTGGAGTCTCA

CGGAGACTCTGCATATTGGTCAGCTCAGTATTAACTTATTCGGTGAGTGC

TGTCACCAGATCTCGTCCCGCCTGCATTCCCAGGGCTTGCAGCGACATTG

AGGCATCTGCCCGCCTGTCCGACCACCCGGGAGGGGGGTAAGATTTGAGA

GGTACTTTATAGGGGCAGTTAAATGAAGACGCAAACAAGTCCTAGTGTTG

ATGCGGAACTGCGCGCCGAATGCCTTGGCTCTGACACCTGTTGAGCTGCA

GGACTCCGCTAAAGCGTCCCACCTAATGACTGTAACAACGTCCCCTGAGG

AGGGCCAATATGGCGACGGTCTCCTCTTGGCATAGCCCTCTTCCCTCCCT

CATGATGGGCAGCTCCAGTAACGCCCATTGGCTAACTAGGAGGCGGTGCC

AGGCCTACTTCGTCCCCTCATTGGATTGAATAACTGAGGGAGCCGCCAAT

TCTCCTCTGCCACTCCAAGTTTCCGCCCTCAGTTAATTCGGCGTTTAATT

GGCTTTTAGTTCACGTCAATATGCGTCCTTTCCTGTCTCTTTTCAGTCTA

ACTCCAATCATAACGTTCCTGGCTGCCCGCCTGATTTCTGATTGGTTTTA

ATCAGCTTCATCCTCTCCTATTCCTGCCTACTTCTTACCTCTCCGCCCAC

TAGGATTTTGCCCAAGCATATCCCGGATTCTGGTTGGCCGTTGTTCTGTC

ATTCCTATCAAAGCTTTGCCTATCCCTACGTCTCAGGGAGCCCGCCTGCC

GGTTGACTGGTTTCCTTCCAAGCCAATCATCTCCAGCTCCCGCCCATCTT

CACTTCCTGCATCCTTCATTGGCTTTTAACACTGAGAGGGCGGTCTTTTT

AGGCGGACACCAGGCACGCAACTTAGTCTCACACGCCTTGGAGAGCAAGC

GAGTCTTGCCATTGGATAATTCCACCGTCTTTCTTCTGCAAGTCCCTCCT

TTCCCCCTCCCTCATTGGGCGGGGCAGCAGAGAAGGGGCGGGGCCTAGGT

TGGGCTTGTGGCGCGCTGCTCCCTCCTCCTTACCCCCCCCTCCCTGTCCG
